# Supra hepatic inferior vena cava and right atrial thrombosis following a traffic car crash

**DOI:** 10.5249/jivr.v8i2.769

**Published:** 2016-07

**Authors:** Feridoun Sabzi, Hosein Karim, Marjan Haghi

**Affiliations:** ^*a*^Department of Cardiovascular Surgery, Imam Ali Heart Center, Kermanshah University of Medical Sciences, Kermanshah, Iran.; ^*b*^Department of Gerontology, University of Social Welfare and Rehabilitation Sciences, Tehran, Iran.

## Abstract

We present a case of nephrotic syndrome associated with right atrial and supra hepatic vein part of inferior vena caval thrombosis. This patient presented with dyspena, lower extremity edema and back pain after a vehicle accident and blunt trauma to the abdomen. Trauma should be considered not only as a thrombophilic pre-disposition, but also as a predisposing factor to IVC endothelium injury and thrombosis formation. Echocardiography revealed supra hepatic vein IVC thrombosis floating to the right atrium. A C-T scan with contrast also showed pulmonary artery emboli to the left upper lobe. With open heart surgery, the right atrial and IVC clot were extracted and the main left and right pulmonary arteries were evaluated for possible clot lodging. The patient had an uneventful postoperative recovery and thrombosis has not reoccurred with periodical follow-up examinations.

## Introduction

The predisposing factors for thrombosis of the inferior vena cava (IVC) have similar etiological factors to lower extremity deep venous thrombosis (DVT). ^1^ The vircho triad for clot formation may be related to trauma (endothelial injury), thrombophilic state, hematological or neoplastic abnormalities, extra luminal pressure to IVC (tumor, hepatic abscess, iatrogenic stenosis) caused venous stasis that have all been implicated as primary mechanisms in the path physiology of IVC thrombosis. ^2^ We present a case of traumatic IVC thrombosis in a previously healthy young man, who presented with right atrium and supra hepatic vein IVC thrombosis and pulmonary emboli thought to be secondary to a trauma-induced IVC endothelial injury and secondary nephritic syndrome.

## Case report

**Figure 1:**
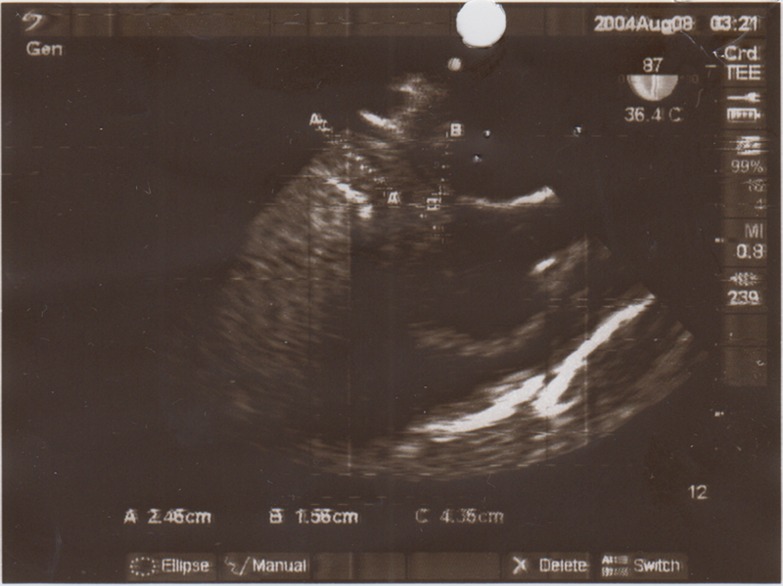
Echocardiography view showed thrombosis in the entrance to the inferior vena cava to the right atrium.

**Figure 2:**
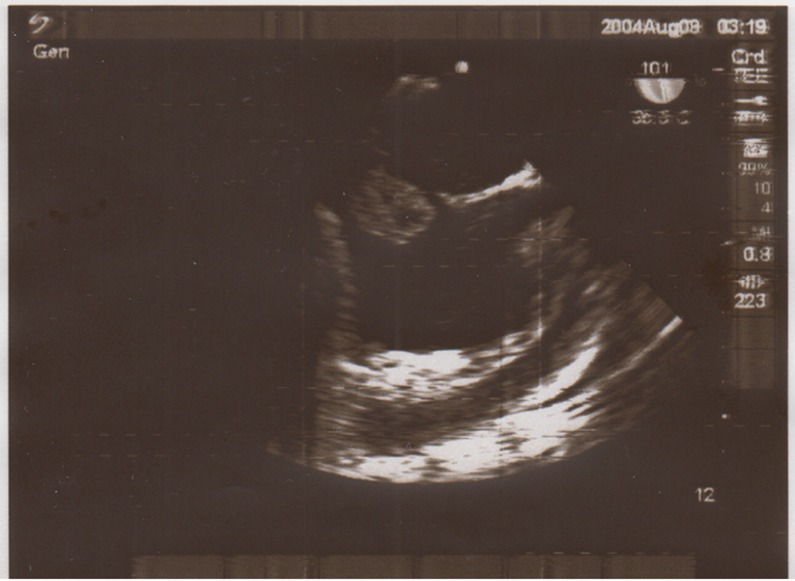
Transthoracic echocardiography M Mode view revealed a large clot in the entrance of inferior vena cava to the right atrium.

**Figure 3:**
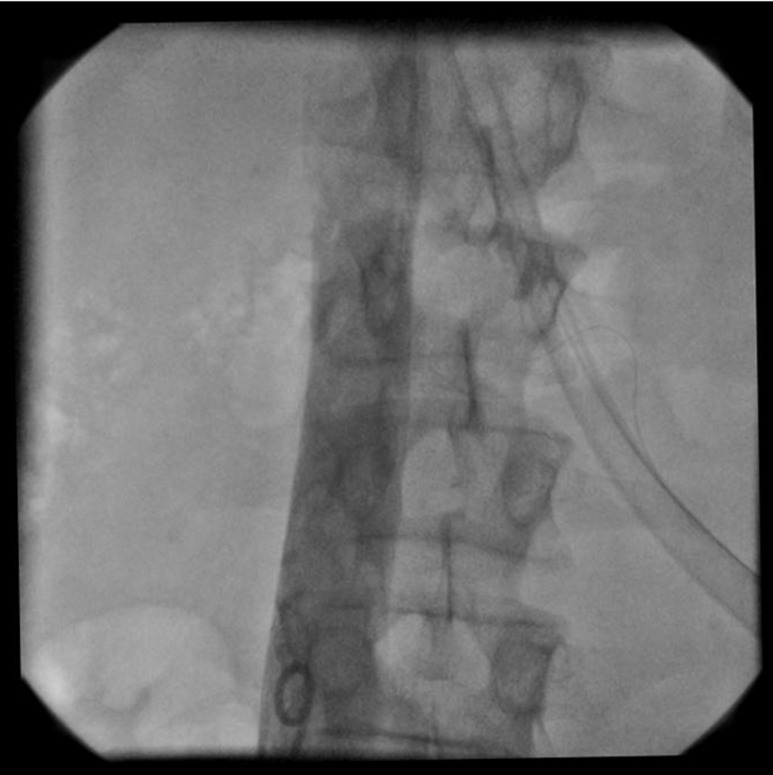
Patency of abdominal vena cava in vena cavography.

**Figure 4:**
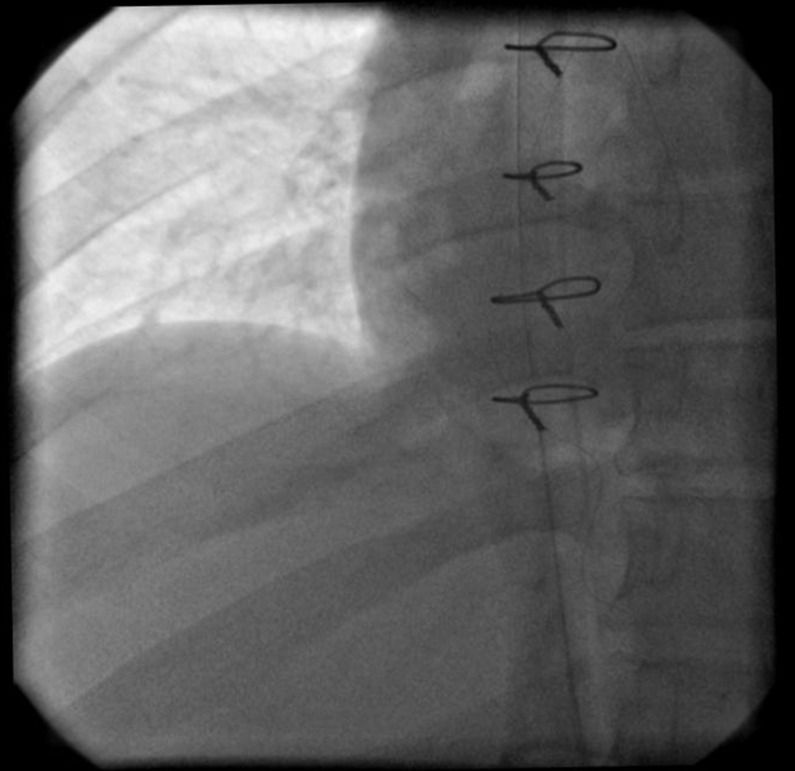
Vena cavography shows patency of supra hepatic vein and right atrium

A 30-year old male truck driver presented in our center with a 10-day history of abdominal crash injury followed by secondary lower back pain, lower extremity pitting edema, scrotal edema, dyspnea and a temperature of 38°C. On examination, he was hemodynamically stable and his abdomen was soft with mild right upper quadrant abdominal tenderness. Hematological analysis confirmed no anemia and the lactate dehydrogenase level was normal and the C-reactive protein was markedly elevated at 190 mg/L. All other hematological, coagulation, thrombophilic tests and biochemical analyses were normal. Bilateral lower limb arterial examination was normal. Plain X-rays of the chest, abdomen and lumbar spine were normal. Laboratory results also showed urea (23 mg/dl), creatinine (1.2 mg/dl), sodium (136 mmol/L), and potassium (3.6 mmol/L) that were normal. Urinalysis, using a urine dipstick, revealed three pluses of proteinuria, which equates to ≥3g urinary protein per day. Lab examination for the measurement of serum albumin revealed hypoalbuminemia. Doppler ultrasound of the femoral veins demonstrated marked expansion of both vessels without intra-luminal thrombus. A trans-thoracic echocardiography revealed a free floating mass in the IVC entrance extended to the right atrium (Figure 1 and 2). Poor view abdominal sonography revealed a probable clot in the supra hepatic IVC but the IVC and bilateral iliac veins were distended and no tumor was found in liver or retroperitoneal spaces. The patient was scheduled for emergency open heart surgery. Median sternotomy and vertical pericardiotomy were performed. Cardiopulmonary bypass (CPB) was established with aortocaval cannulation. The pulmonary artery was dissected and clamped. After cooling to 20°C, cardioplegic arrest was achieved with cold blood cardioplegia. Total circulatory arrest was established. The right atrium (RA) was opened parallel to the atrio-ventricular groove. The thrombus was extracted. The IVC was thoroughly irrigated with normal saline. The circulation was temporarily restarted to flush out any small remnant of the thrombus in the IVC. The venous cannula was advanced into the SVC and the SVC was snuggled. The main pulmonary artery was opened and the left and right pulmonary artery suctioned and a large clot was found in the left pulmonary artery. Circulation was restarted and the RA was closed. CPB was weaned off after re-warming. The perioperative course was uneventful. The CPB and aortic cross-clamp times were 55 and 21 minutes, respectively. Total circulatory arrest time was 5 minutes. With continuation of lower extremity edema and signs of nephrotic syndrome, an IVC venogram via the femoral vein in the post-operative period demonstrated patency of the IVC inferior to the right atrium and patent renal veins and hepatic veins (Figure 3 and 4). Anticoagulation with warfarin was started on the day following surgery and continued for 6 weeks. At the four month follow up, there was no evidence of recurrent thrombosis in the hepatic vein, IVC or RA or proteinuria. It was felt on balance that treatment should be directed towards the thrombus. His bilateral lower limb pain and edema resolved at an early stage and the patient remained well two months later with regular vascular and hematological clinical review.

## Discussion

**Hodkinson** found that thrombosis of the IVC and subsequent right atrial clot is an exceedingly rare complication of trauma. The exact pathogenesis of IVC thrombosis in association with trauma is uncertain. The trauma process in the abdomen may cause injury to the intima of the IVC, leading to inflammation followed by thrombosis. The process is usually initiated in the junction of the IVC and the hepatic vein from where the thrombus extends into the IVC and may reach up to the RA.^3^ **Giordano** found that the extension of post-traumatic thrombus into the RA is rare and there have been only a few published case reports in the literature. ^4^ **Anderson** demonstrated that although the overall incidence of venous thrombosis is 0.5%, it still remains a rare condition especially in young patients. ^5^ Our patient had two important risk factors, namely endothelial injury and thrombophilia induced by nephrotic syndrome. **Jackson ** showed that the clinical presentation of IVC thrombus varies according to the extension of the clot to right atrium, emboli to pulmonary artery, the level of the thrombosis, involvement of hepatic vein, renal vein lower extremity and some patients remain asymptomatic. Various levels of thrombosis cause a variety of signs and symptoms such as lower extremity edema, scrotal edema back pain, proteinuria, hepatic engorgement, cardiac failure and pulmonary embolus. ^6^ **Tsuji ** found that IVC clotting caused an inflammatory reaction in half of the patients who presented with pyrexia and elevation in d-dimer levels and inflammatory markers (white cell count, C-reactive protein). ^7^**Cellarier** found that post-traumatic thrombotic events are exceptional in the caval system and reported a case of inferior vena cava thrombosis in a traffic accident victim. Intimal injury as a predisposing factor for IVC thrombosis has a different outcome with respect to blunt or penetrating rupture of the IVC.^8^

## Conclusion

A high index of suspicion is warranted for IVC thrombus in post-traumatic patients with lower extremity swelling, dyspnea and concurrent rise in inflammatory markers and pyrexia. In these cases, further investigational modalities are mandatory following open heart surgery. MRI imaging or cavography is required to delineate IVC anatomy and ascertain the distal extent of the thrombus. 
